# Supplementary material for: The macroeconomic effects of adapting to high-end sea-level rise via protection and migration
Source: Nat Commun. 2022 Sep 29;13:5705. doi: 10.1038/s41467-022-33043-z (PMC9522673; doi:10.1038/s41467-022-33043-z)
Supplement: Supplementary file 1 — Supplementary material [file 41467_2022_33043_MOESM1_ESM.pdf]

The macroeconomic effects of adapting to high-end sea-level rise via protection and migration

Supplementary Information

Supplementary Methods 2
COIN-INT model description 2
Regional and sectoral aggregations 2
Specification of production sectors, final demand and foreign trade 4
Dynamics 5
Baseline calibration 6
Additional figures 11
Migrants per year 11
Length of protected coastlines 14
Sea flood costs 17
Migration costs 20
Further results 23
References 28

Supplementary Methods

COIN-INT model description

Regional and sectoral aggregations

Supplementary Table 1. **COIN-INT model region aggregates.**

**Aggregated region**	**Model code**	**GTAP9 regions contained within aggregate**
Germany	DEU	Germany
Austria	AUT	Austria
Italy	ITA	Italy
UK	UKD	UK
France	FRA	France
Belgium and Luxemburg	BLU	Belgium, Luxemburg
Netherlands	NLD	Netherlands
Central EU 27 + Switzerland	CEU	Czech Republic, Hungary, Poland, Slovenia, Slovakia, Switzerland
Northern EU 27+ Liechtenstein, Norway and Iceland	NEU	Sweden, Ireland, Denmark, Finland, Norway, Estonia, Latvia, Lithuania, Rest of EFTA (*Liechtenstein, Iceland*), Rest of the world (*Antarctica, French Southern Territories, Bouvet Island, British Indian Ocean Territory*)
Mediterranean and South-eastern EU 27	MEU	Cyprus, Greece, Malta, Spain, Portugal, Bulgaria, Croatia, Romania, Albania, Rest of Europe (*Bosnia and Herzegovina, Macedonia, Serbia and Montenegro, Faroe Islands, Gibraltar, Monaco, San Marino*)
North America	NAM	USA, Canada, Rest of North America (*Bermuda, Greenland, Saint Pierre and Miquelon*)
Australia and New Zealand	AUZ	Australia, New Zealand
Eurasian countries	ERA	Russian Federation, Kazakhstan, Belarus, Ukraine, Armenia, Georgia, Kyrgyzstan, Rest of former Soviet Union (*Tajikistan, Turkmenistan, Uzbekistan*), Rest of Eastern Europe (*Moldova*)
Emerging economies- Asia	ECA	Hong Kong, Singapore, South Korea, Japan
Turkey	TUR	Turkey, Israel
China	CHN	China
India	IND	India
South-East Asia	SEA	Bangladesh, Thailand, Indonesia, Vietnam, Pakistan, Tunisia, Malaysia, Taiwan, Philippines, Cambodia, Lao People's Democratic Republic, Rest of South-East Asia (*Myanmar, Timor-Leste*), Sri Lanka, Rest of South Asia (*Afghanistan, Bhutan, Maldives*), Rest of East Asia (*Korea, Macau*), Rest of Oceania, Nepal, Brunei Darussalam, Mongolia
Latin America (w/o Venezuela)	LAM	Brazil, Mexico, Argentina, Bolivia, Guatemala, Honduras, Nicaragua, Peru, Rest of South America, Chile, Colombia, Dominican Republic, Ecuador, El Salvador, Paraguay, Uruguay, Costa Rica, Panama, Rest of Central America, Trinidad and Tobago, Caribbean (*Anguilla, Antigua and Barbuda, Aruba, Bahamas, Barbados, British Virgin Islands, Cayman Islands, Cuba, Dominica, Grenada, Haiti, Montserrat, Netherlands Antilles, Saint Kitts and Nevis, Saint Lucia, Saint Vincent and Grenadines, Turks and Caicos Islands, Virgin Islands (US)*), Jamaica, Puerto Rico

Aggregated region	Model code	GTAP9 regions contained within aggregate
Oil exporting countries (OPEC: Middle East and Africa + Venezuela)	OIE	Saudi Arabia, United Arab Emirates, Egypt, Nigeria, Venezuela, Rest of North Africa (*Algeria, Lybia*), Rest of Western Asia (*Iraq, Lebanon, Palestinian Territory, Occupied, Syrian Arab Republic (Syria), Yemen*), Azerbaijan, Iran, Bahrain, Kuwait, Oman, Qatar, Morocco, Rest of South Central Africa, Jordan
Africa	AFR	South Africa, Benin, Burkina Faso, Cameroon, Cote d'Ivoire, Ghana, Guinea, Senegal, Togo, Tunisia, Rest of West Africa, Central Africa, Ethiopia, Kenya, Madagascar, Malawi, Mauritius, Mozambique, Namibia, Rwanda, Tanzania, Uganda, Zambia, Zimbabwe, Rest of Eastern Africa, Botswana, Rest of South African Customs Union

Supplementary Table 2. **COIN-INT model sector aggregates**. Superscripts 1-5 denote attribution to one of the five groups: 1. Resource using sectors, 2. Agricultural sectors, 3. Refined petroleum and coal products, 4. Process-emission generating sectors, 5. Non-resource using sectors.

Acronym	Sector aggregates in the model	GTAP9 sectors contained in aggregate
AGC [2]	Agricultural products -crops	Agricultural sectors (1-8)
AGL [2]	Agricultural products -livestock	Agricultural sectors (9-12)
FOF [1]	Forestry and Fishery	forestry (13) and fishing(14)
COA [1]	Coal	Coal Mining (15)
OIL [1]	Crude Oil	Oil extraction (16)
GAS [1]	Natural Gas	Natural Gas extraction (17), manufacture of gas, distribution, steam and hot water supply (44)
OMN [1]	Other mining	other mining (18)
ELY [5]	Electricity	Production, collection and distribution of electricity (share of 43)
FBT [5]	Foodstuffs and feedingstuffs, beverages and tobacco products	All food processing sectors (19-25), beverages and tobacco products (26)
TWO [5]	Textile industry and other manufacturing	Textiles (27), Wearing apparel (28), Leather products (29), Wood products (30), Manufacture of paper products and publishing (31), Other Manufacturing: includes recycling (42)
OME [5]	Machinery, data processing equipment, electronic and optical products, Electronic Equipment, Motor, Motor vehicles and parts and other Transport Equipment	Other Machinery & Equipment: electrical machinery and apparatus n.e.c., medical, precision and optical instruments, watches and clocks (41), Electronic Equipment: office, accounting and computing machinery, radio, television and communication equipment and apparatus (40), Motor, Motor vehicles and parts: cars, lorries, trailers and semi-trailers (38), Other Transport Equipment: Manufacture of other transport equipment (39)
P_C [3]	Refined oil products	Petroleum, coal products (32)
CRP [4]	Chemical industry	Chemical, rubber, plastic products (33)
MIS [4]	Manufacture of other non-metallic mineral products, precious and non-ferrous metals, of basic iron and steel and casting and fabricated metal products	Manufacture of other non-metallic mineral products (34), precious and non-ferrous metals (36), Manufacture of basic iron and steel and casting (35), fabricated metal products (37)
WAT [5]	Transport –Water	Water (49)

Acronym	Sector aggregates in the model	GTAP9 sectors contained in aggregate
AIT [5]	Transport –Air	Air (50)
LAT [5]	Transport – Land	Other Transport (including road and rail transport) (48)
SER [5]	Other services and utilities	Water (45), Trade: all retail sales; wholesale trade and commission trade; hotels and restaurants; repairs of motor vehicles and personal and household goods; retail sale of automotive fuel (47), financial services (52), post and telecom (51), Recreational & service activities (55), dwellings (57) real estate & other business (54)
PIN [5]	Private insurance	insurance (53)
OSG [5]	Public services	other services (government): public administration (56)
CON [5]	Construction	construction (46)

Specification of production sectors, final demand and foreign trade

COIN-INT features a multitude of different production sectors. All of them are implemented as nested constant elasticity of substitution (CES) production functions. These CES functions, however, differ for different groups of sectors to capture key characteristics of production technologies (see Schinko et al. for details[1]). These groups are:

1. **Resource using sectors**: Coal Extraction, Oil Extraction, Gas Extraction, Other Mining, Forestry & Fishery. These sectors all use limited natural resources to operate. These sectors thus provide raw materials to the market.
2. **Agricultural sectors**: Agricultural crop and agricultural livestock sectors, using land as a production factor. Land is exclusively used by these two sectors.
3. **Refined petroleum and coal products**: This sector aggregate uses fossil raw materials to produce refined fossil fuels (essentially oil and coal).
4. **Process-emission generating sectors**: Sectors that emit process-emissions, i.e., greenhouse gas emissions that do not originate from the combustion of fossil fuels but stem from chemical processes in the course of production. This group comprises the Iron & Steel sector, non-metallic minerals (including cement) as well as the chemical industry.
5. **Non-resource using sectors**: This groups comprises all remaining sectors.

In addition to the described sectoral structure, the Electricity sector is further divided into 12 sub-sectors according to the GTAP-POWER database[2]. The 12 sub-sectors represent seven base-load

electricity generation technologies (Nuclear, Coal, Gas, Wind, Hydro, Oil, Other) as well as four peak-load technologies (Gas, Hydro, Oil, Solar). In addition, there is a transmission and distribution-sector.

Final demand is generated as follows: Within the EU there are two representative households in each region. First, a private household which is endowed with the production factors skilled labour, unskilled labour, capital as well as natural resources (fossil resources, land and CO_2 emission allowances). These factors and resources are provided to the market and thus create income. The resulting income is spent for either consumption or investment (savings), subject to a fixed savings rate. Private consumption is specified as a nested CES consumption function. Second, in each EU region there is also a public household, which collects taxes and provides transfers to the private household. Net-tax income is used to finance the supply of public services, i.e. government consumption, which is specified as a Leontief consumption function (i.e. fixed expenditure shares within the consumption bundle). Tax rates are assumed to be fixed; thus, government income is flexible. By default, transfers to the private household are assumed to scale linearly with tax income.

Foreign trade is implemented according to the Armington assumption[3]. Hence, each region treats domestically produced goods and imported goods differently, subject to sectorally differentiated elasticities of substitution from GTAP9. Foreign trade is closed by assuming a fixed current account balance, which grows with GDP. The current account is balanced via net-capital inflows of opposite sign (i.e., the capital account).

Dynamics

COIN-INT is available in two variants: a static comparative version which models snapshots of 2011, 2030 and 2050, as well as a recursive dynamic version that explicitly models the pathway of economic development in 5-year times steps from 2015 to 2050.

In the comparative static variant the supply of production factors and resources is exogenously scaled up according to the quantitative indicators of the Shared Socioeconomic Pathways (SSPs; see O’Neill et al. for details[4]). Total factor productivity is determined endogenously to meet the SSP-specific GDP growth rates.

In the dynamic recursive variant time steps are modelled explicitly, which are connected via the following equation of capital accumulation: $K_{t+1} = K_t*(1-\delta) + I_t$. This equation reads as follows. The capital stock (K) of the next year period (t+1) is determined by the current year (t) capital stock, minus deprecation according to the depreciation rate (δ), plus current period Investments (I). The recursive dynamic model specification implicitly assumes myopic behaviour of all economic agents, that is, they do not include future expectations in their decision but optimize within the current period. Depreciation rates and 2011 regional capital stocks are taken from GTAP9. In order to prevent very strong/weak capital accumulation until 2050 due to possible high/low investment levels in the benchmark year 2011, the savings rate is assumed to converge in all regions to 25% until 2050[5]. The endogenously derived capital stock thus drives the availability of the production factor capital (i.e. the annual capital rent of the capital stock). The availability of labour is given exogenously via the growth of the working age population in each region, according to SSPs. Total factor productivity is determined endogenously to meet the SSP-specific GDP growth rates. Land availability is exogenous, according to SSPs. Natural resource supply (including fossil fuels) is determined endogenously.

Baseline calibration

COIN-INT is calibrated to the RCP-SSP framework. The calibration process involves two steps.

In **step 1** the SSP-Baselines are calibrated, i.e., socio-economic developments without any additional climate policy as given in 2011 are constructed. The socio-economic development is driven by exogenously given population growth, endogenous total factor productivity (TFP) changes, exogenously given autonomous energy efficiency improvements (AEEI, based on Dai et al.[6]) as well as exogenously given electricity generation cost digressions. Fossil fuel supply is set endogenously to meet the SSP-specific fossil fuel price forecast (for SSP2 this is based on IEA[7]). In step 1 SSP2 is selected as a central case and deviations from SSP2 parameters are introduced for calibrating other SSPs (i.e., SSP1, SSP3 and SSP5). Parameters are set in a way such that the CO_2 emissions calculated by COIN-INT come close to the IIASA SSP marker-scenarios' CO_2 emissions[8]; see Supplementary Figure 1 (note that CO_2 emissions from land use changes are not included in the model). For a full overview on the parameter settings of the SSP-Baseline calibration see Supplementary Table 3.

In **step 2** the SSPs are combined with RCPs by introducing a set of "Shared Policy Assumptions" (SPAs). These are: CO_2-pricing, subsidies for climate neutral Iron & Steel production (for sector MIS) as well as standards (which are assumed to increase the share of renewable electricity as well as flexibility in power generation and consumption). Note, that in the calibration process the CO_2-price is determined endogenously such that the exact emission pathway as given by the RCPs are reached. Emission reduction obligations are set exogenously for each region. The reduction targets are determined based on step 1 of the calibration procedure: Global emissions from step 1 are used to calculate a global relative emission reduction requirement to meet the given RCP. The same relative emission reduction requirement is then applied to all model regions uniformly. This means that each region fulfils the same relative emission reduction in a future year (relative to the respective Baseline emissions from step 1). The endogenously derived CO_2 prices are regionally different, except for the EU with one shared price and emission allowance framework. Supplementary Figure 2 shows the global average CO_2 prices in 2050 (weighted by regional emissions) for all modelled SSP-RCP-combinations. As expected, CO_2 prices need to be higher, the lower the RCP, and need to be lower in more sustainable SSPs.

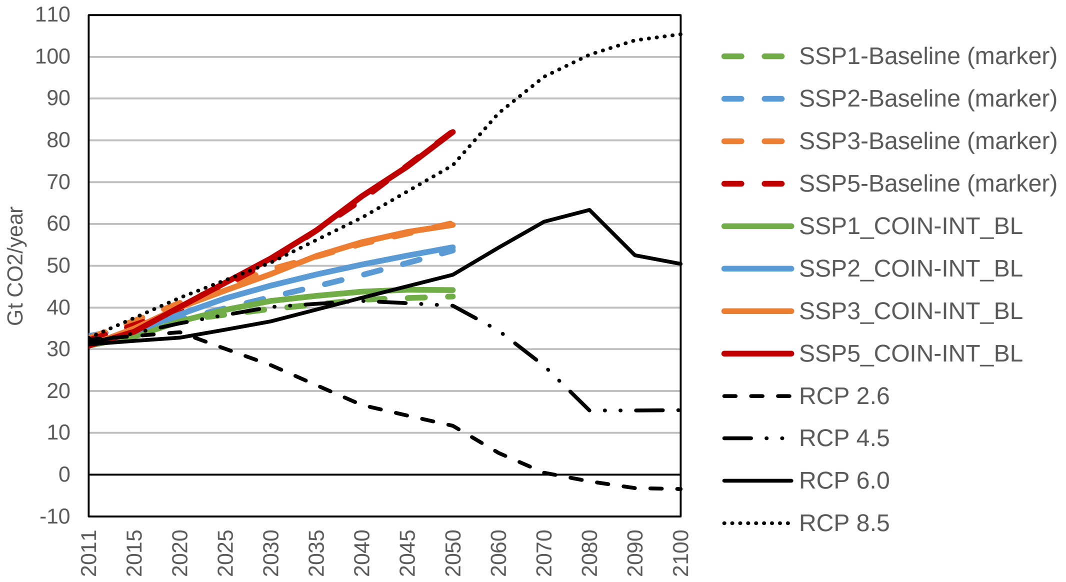

Supplementary Figure 1. **Comparison of CO_2 emissions of the COIN-INT Baseline scenarios and SSP marker-scenarios.**

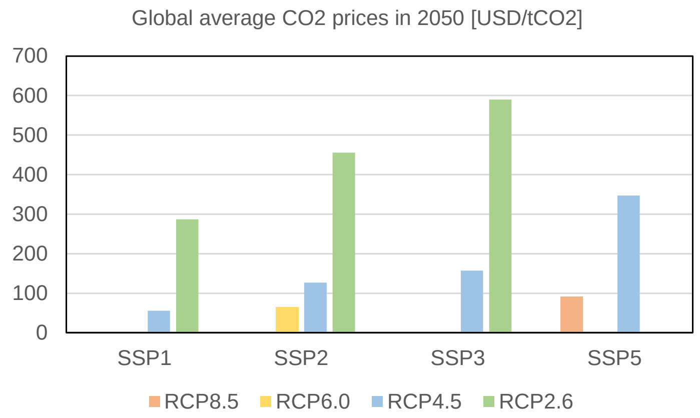

Supplementary Figure 2. **Global average CO_2 prices for all calibrated SSP-RCP combinations.** Weighted by regional emissions.

Supplementary Table 3. **SSP calibration parameter settings in COIN-INT.**

	**Sustainability—Taking the green road**	**Middle of the road**	**Regional rivalry—A rocky road**	**Fossil-fueled development—Taking the highway**
**Parameter**	**SSP1**	**SSP2**	**SSP3**	**SSP5**
Fossil fuel price forecast	=SSP2 (Actually no reason for exogenously increasing/decreasing the given fossil fuel price forecast. In SSP1 demand for fossil fuels is lower than in SSP2. If supply would not react, this would lead to lower prices, however in such a sustainable world supply should also adjust (i.e. decrease) due to expectations of the suppliers.)	following IEA[7] "current policies scenario" until 2050:<br>Coal: -0.3% p.a.<br>Oil: +1.5% p.a.<br>Gas: +1.4% p.a.	>SSP2, due to high demand: +0.5% p.a.	>>SSP2, due to high demand: +1% p.a.
AEEI (in non-ELY sectors)	>SSP2: +1% p.a.	2-5% p.a., depending on region (developed versus developing) and energy type (based on (Dai et al., 2017)	<<SSP2:<br>developed regions: -1% p.a.<br>Developing regions: -2.5% p.a. (gas: 0%)	<SSP2:<br>developed regions: -0.5% p.a.<br>Developing regions: -0.5% p.a.
Electricity generation cost digression p.a. (on all non-energy intermediate inputs and production factors)	>SSP2: +1% p.a. for all non-fossil ELY generation technologies; -2% p.a. for fossil ELY generation (risk markup)	Hydro: 0.25% (i.e. 10% in 2050)<br>Other: 0.47% (i.e 20% in 2050)<br>Wind: 1.79% (i.e. 100% in 2050)<br>Solar-PV: 2.38% (i.e. 150% in 2050)<br>Nuclear: 0.47% (i.e. 20% in 2050)<br>Fossil: 0% (i.e. 0% in 2050)	<SSP2: by 20% lower than SSP2 values to reflect slow technological change (for renewables);<br>for fossils=SSP2	= SSP2 for renewables;<br>0.25% for fossils (i.e. 10% in 2050) to reflect fossil fuel tech-driven world
Change elasticity of substitution within energy bundle	>SSP2: SSP2 * 2 to reflect technological change	default GTAP (0.16)	=SSP2	=SSP2
Change elasticity of substitution within peak load bundle	>SSP2: SSP2 * 5 to reflect technological change	default GTAP-POWER (0.472)	=SSP2	=SSP2
Change elasticity of substitution within base load bundle	>SSP2: SSP2 * 2 to reflect technological change	default GTAP-POWER (1.386)	=SSP2	=SSP2

	Sustainability—Taking the green road	Middle of the road	Regional rivalry—A rocky road	Fossil-fueled development—Taking the highway
**Parameter**	**SSP1**	**SSP2**	**SSP3**	**SSP5**
Elasticity of substitution between base- and peak-load bundles	>SSP2: 2	default GTAP-POWER (0)	=SSP2	=SSP2
Armington elasticity	>SSP2: increased by 1/4 to reflect stronger global integration	default GTAP	<SSP2: reduced by 1/4 to reflect regional rivalry	>SSP2: increased by 1/4 to reflect stronger global integration
Cost-neutral change in private consumption expenditure shares (coal, oil, gas, refined petroleum products, land and air transport, electricity)	coal, oil, gas, p_c: -40%<br>land transport: -40%<br>air transport: -30%<br>electricity: +5%	coal, oil, gas, p_c: -20%<br>land transport: -20%<br>air transport: -20%<br>electricity: +5%	default GTAP expenditure shares	default GTAP expenditure shares
Technological change in iron and steel sector: switch from current technology to EAF (electric arc furnace)	=SSP2	stylized backstop technology for MIS (based on Mayer et al.[9]), which is 25% more costlier than conventional, but needs more capital and electricity instead of coke input	=SSP2	=SSP2
Increase elasticity of substitution between (LK) and E-nest (technological improvement)	>SSP2: +0.7	Based on Koesler & Schymura[10]: between 0.3 and 1.3;<br><br>+0.3	(Koesler & Schymura, 2015): between 0.3 and 1.3;	(Koesler & Schymura, 2015): between 0.3 and 1.3;
Increase elasticity of substitution between energy and non-energy inputs in consumption function of private household (technological improvement and change of lifestyle)	>SSP2: +0.7	default: 0.2	default: 0.2	default: 0.2

Additional figures and tables

Migrants per year

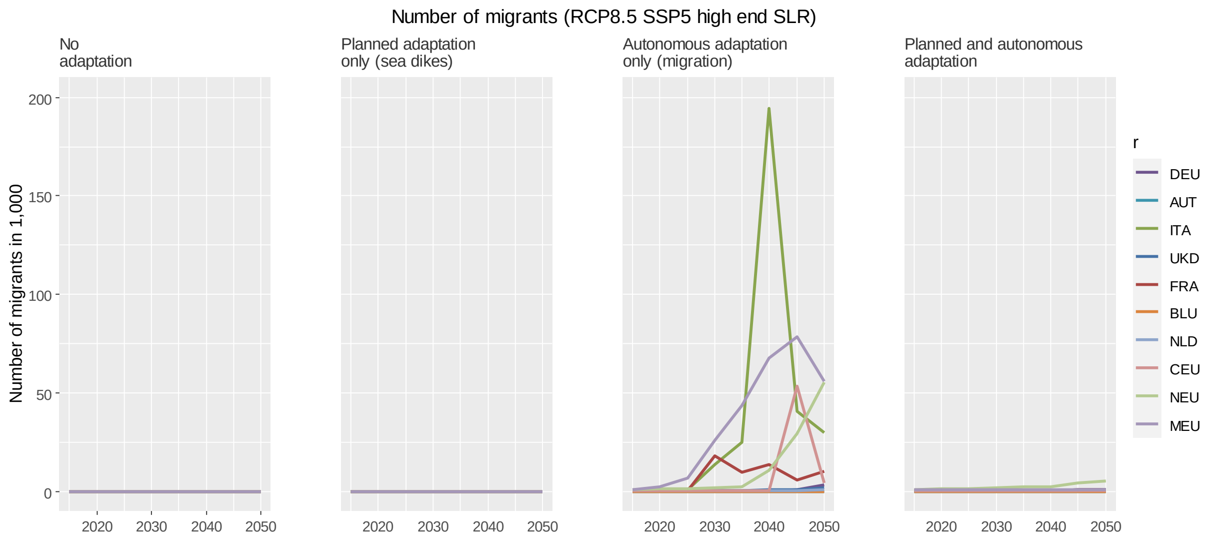

Supplementary Figure 3. **Migrants per year in European regions**. RCP8.5 SSP5 high-end sea level rise, for four cases of adaptation. For region abbreviations, please see Supplementary Table 1.

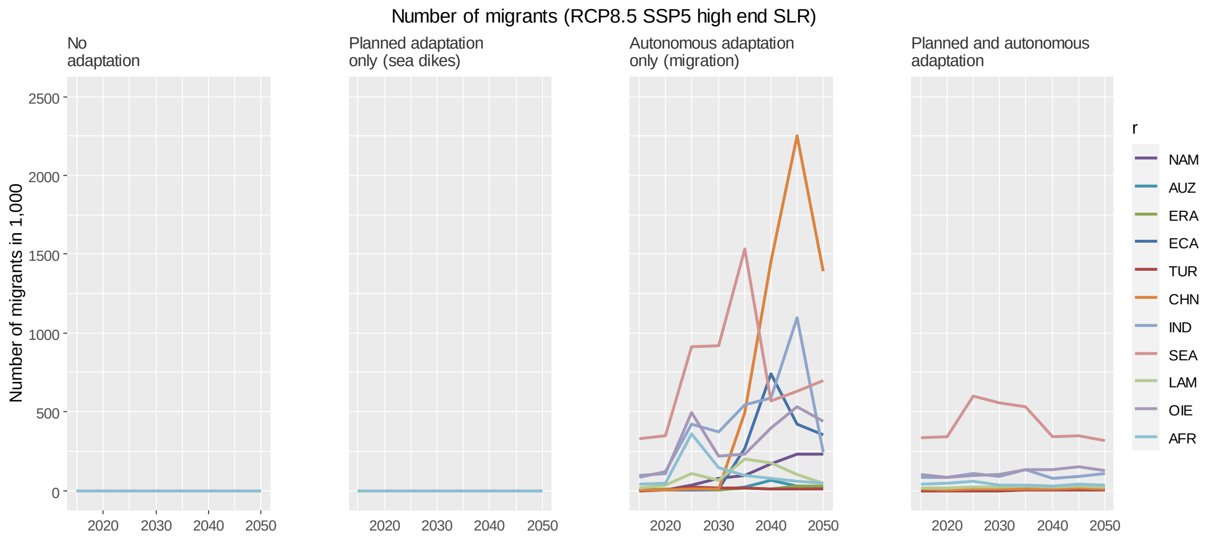

Supplementary Figure 4. **Migrants per year in Rest of the World.** RCP8.5 SSP5 high-end sea level rise, for four cases of adaptation. For region abbreviations, please see Supplementary Table 1.

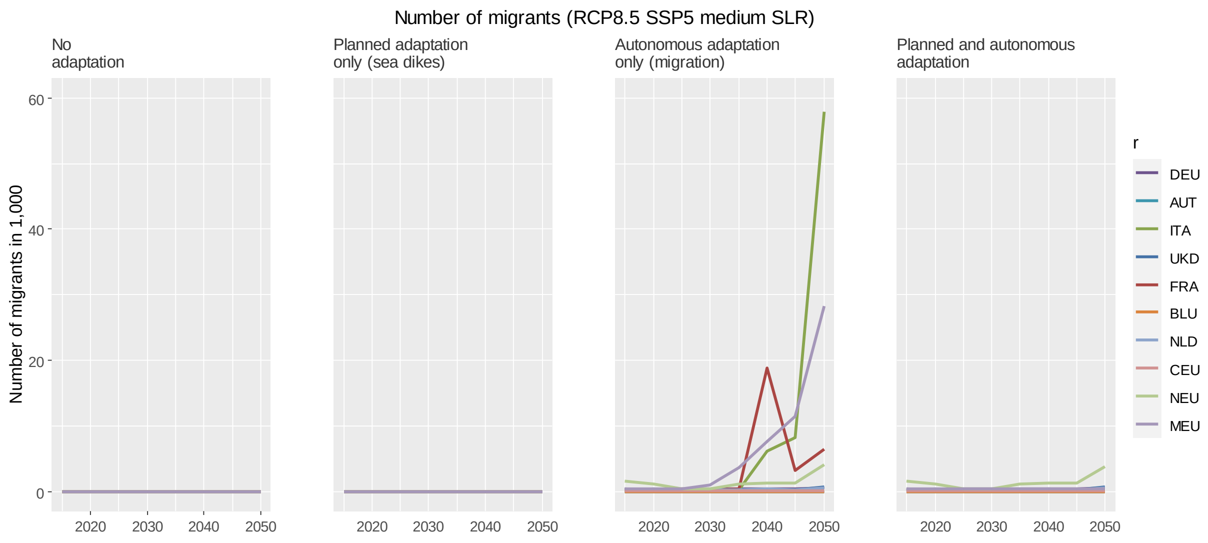

Supplementary Figure 5. **Migrants per year in European regions.** RCP8.5-SSP5 medium sea level rise, for four cases of adaptation. For region abbreviations, please see Supplementary Table 1.

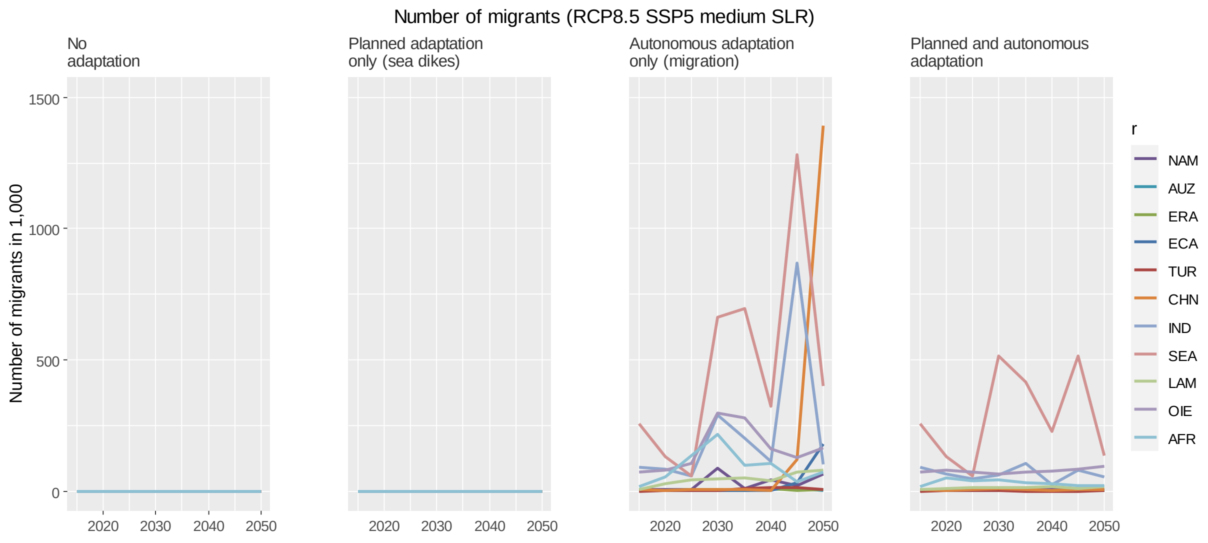

Supplementary Figure 6. **Migrants per year in Rest of the World.** RCP8.5-SSP5 medium sea level rise, for four cases of adaptation. For region abbreviations, please see Supplementary Table 1.

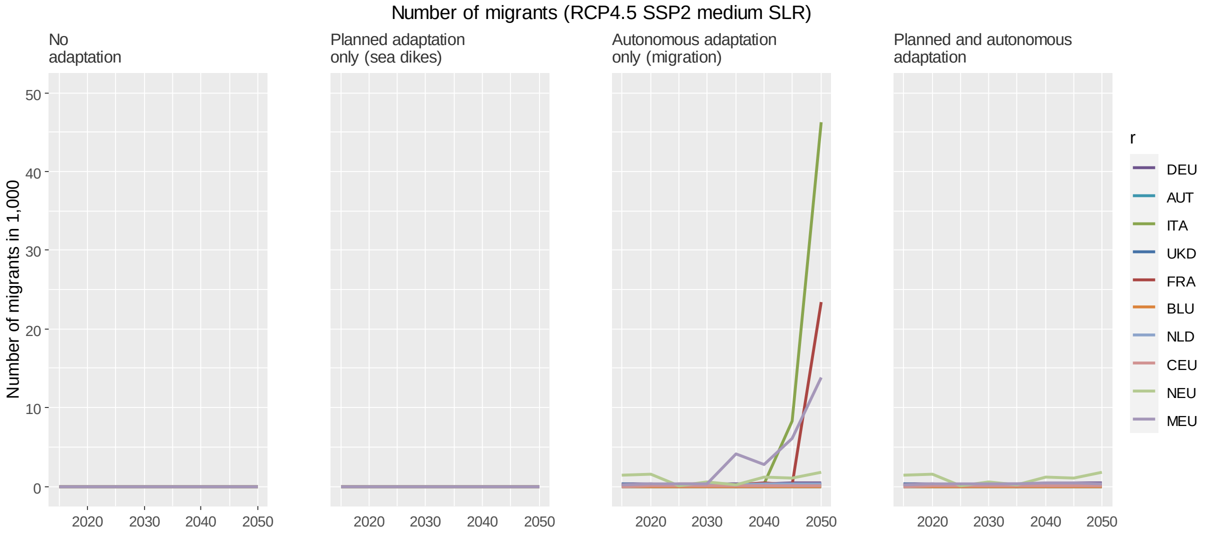

Supplementary Figure 7. **Migrants per year in European regions.** RCP4.5-SSP2 medium sea level rise, for four cases of adaptation. For region abbreviations, please see Supplementary Table 1.

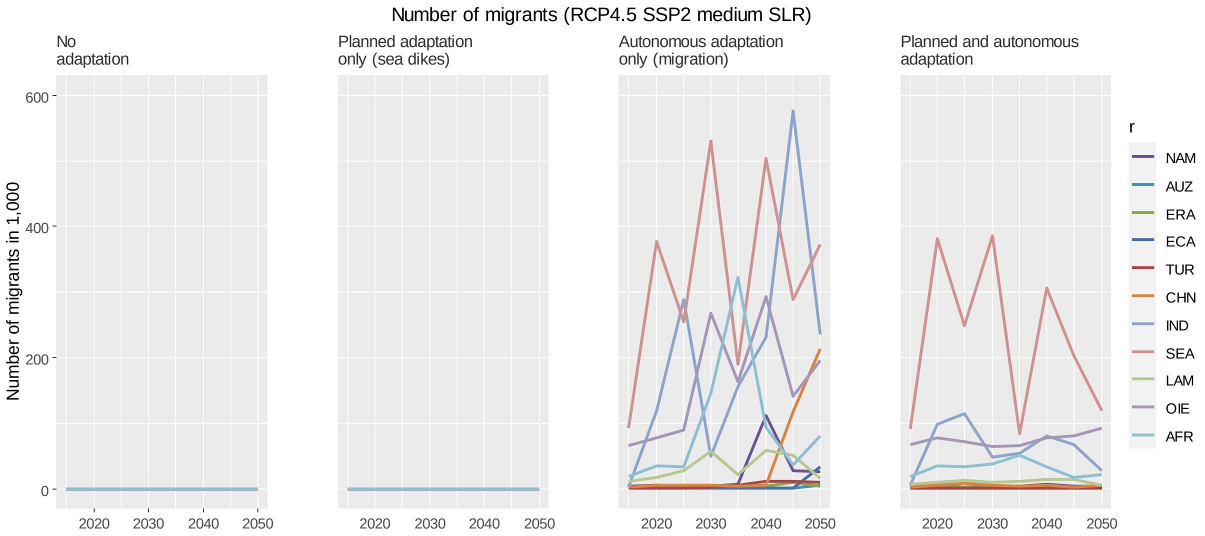

Supplementary Figure 8. **Migrants per year in Rest of the World.** RCP4.5-SSP2 medium sea level rise, for four cases of adaptation. For region abbreviations, please see Supplementary Table 1.

Length of protected coastlines

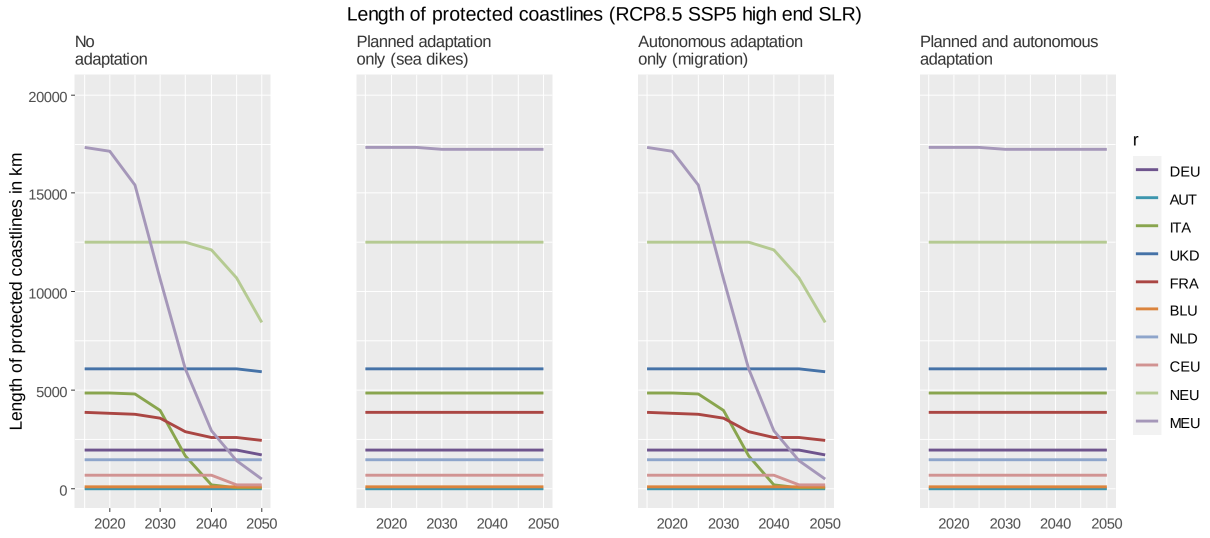

Supplementary Figure 9. **Length of protected coastlines in European regions.** RCP8.5 SSP5 high-end sea level rise, for four cases of adaptation. For region abbreviations, please see Supplementary Table 1.

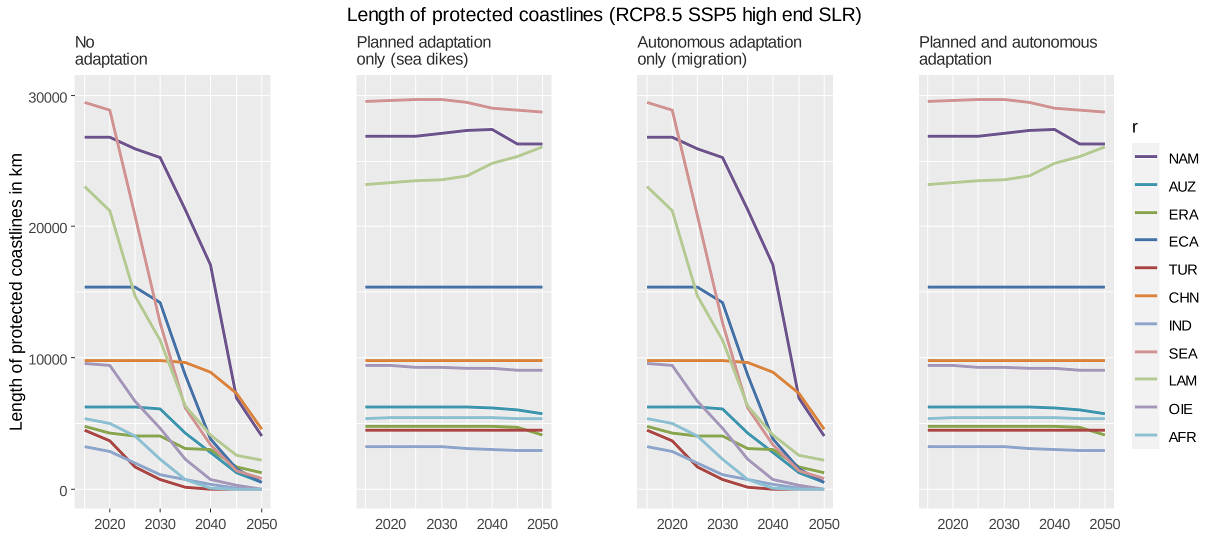

Supplementary Figure 10. **Length of protected coastlines in Rest of the World.** RCP8.5 SSP5 high-end sea level rise, for four cases of adaptation. For region abbreviations, please see Supplementary Table 1.

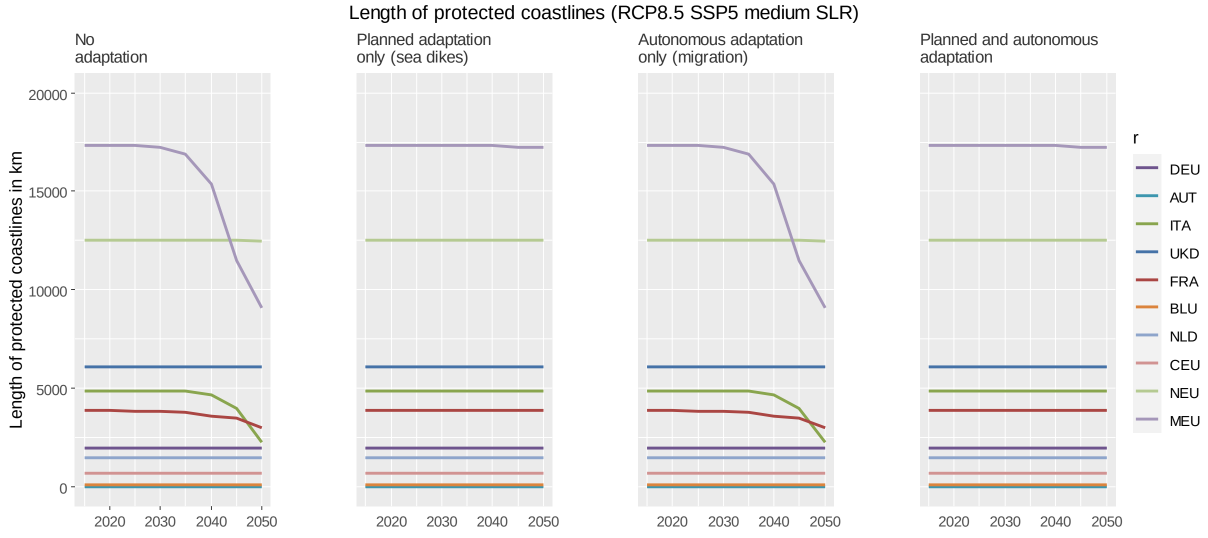

Supplementary Figure 11. **Length of protected coastlines in European regions.** RCP8.5 SSP5 medium sea level rise, for four cases of adaptation. For region abbreviations, please see Supplementary Table 1.

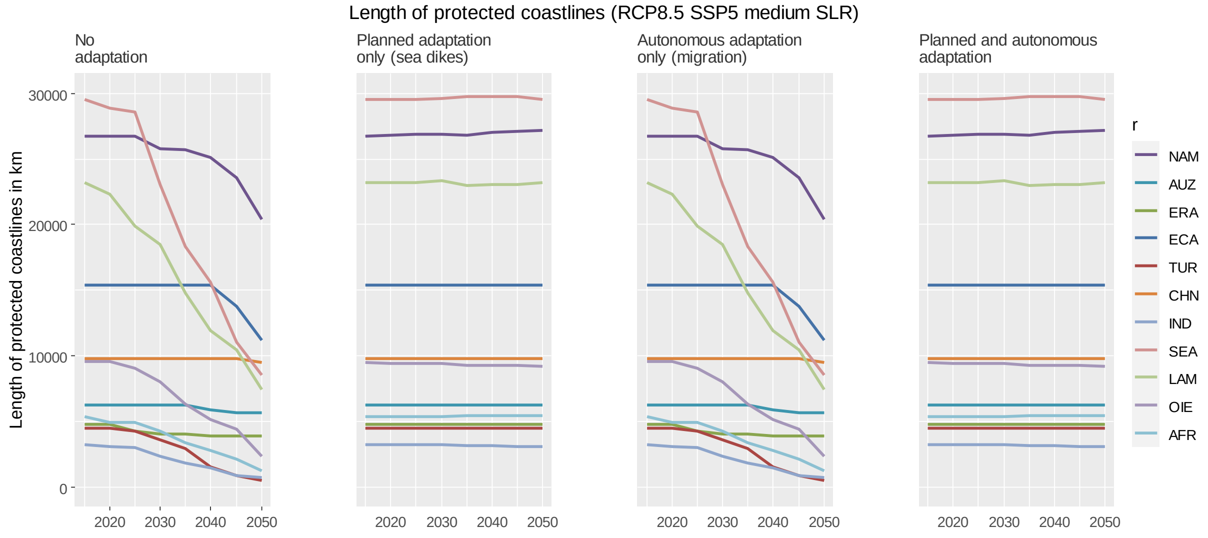

Supplementary Figure 12. **Length of protected coastlines in Rest of the World.** RCP8.5 SSP5 medium sea level rise, for four cases of adaptation. For region abbreviations, please see Supplementary Table 1.

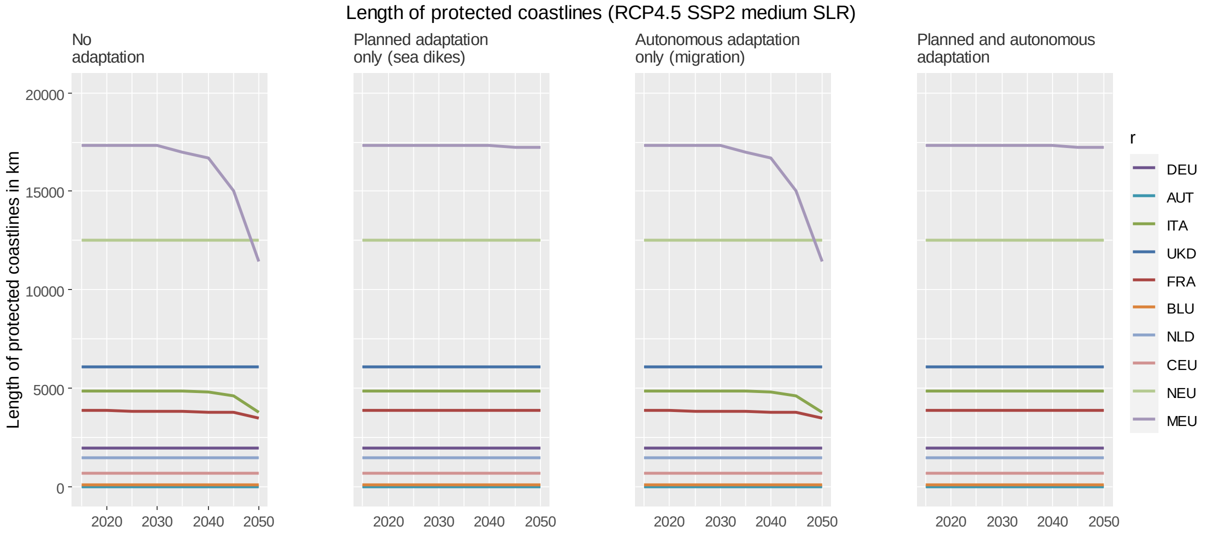

Supplementary Figure 13. **Length of protected coastlines in European regions.** RCP4.5 SSP2 medium sea level rise, for four cases of adaptation. For region abbreviations, please see Supplementary Table 1.

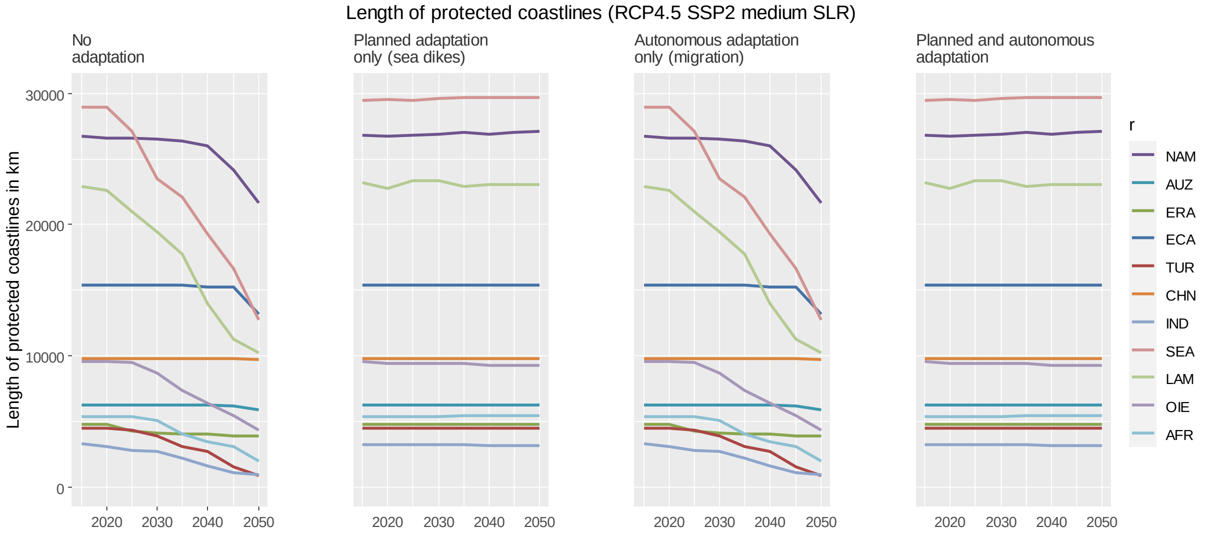

Supplementary Figure 14. **Length of protected coastlines in Rest of the World.** RCP4.5 SSP2 medium sea level rise, for four cases of adaptation. For region abbreviations, please see Supplementary Table 1.

Sea flood costs

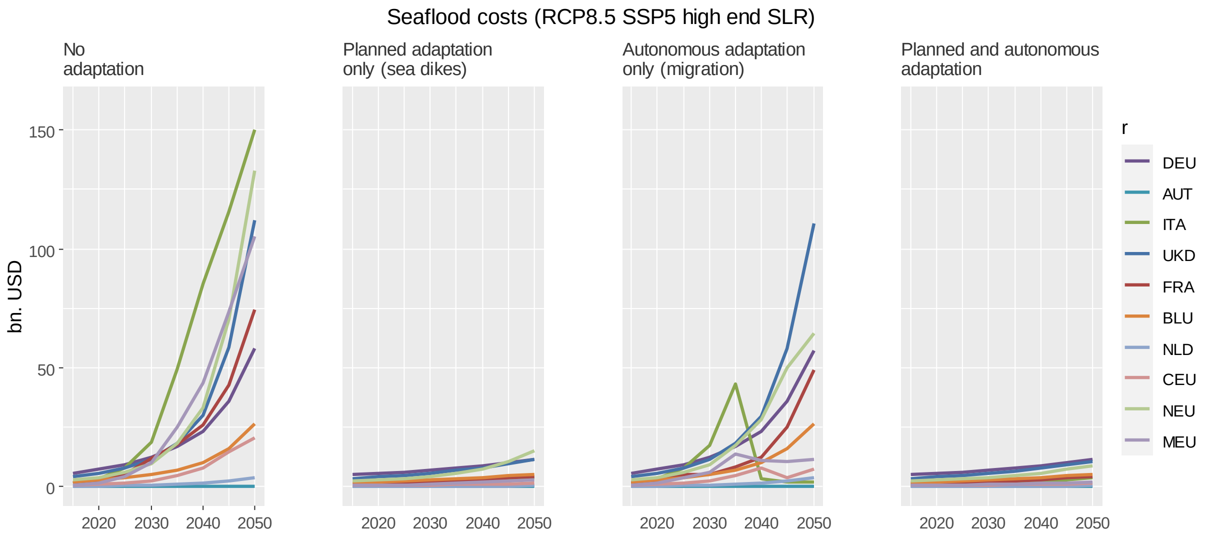

Supplementary Figure 15. **Direct sea flood costs for European regions.** RCP8.5 SSP5 high-end sea level rise, for four cases of adaptation. For region abbreviations, please see Supplementary Table 1.

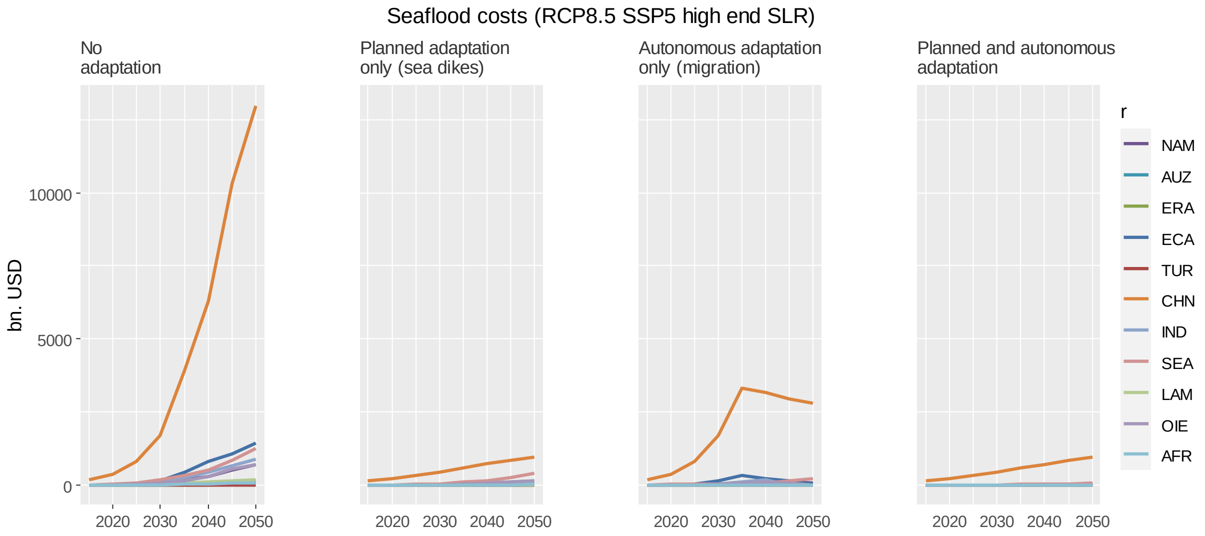

Supplementary Figure 16. **Direct sea flood costs for Rest of the World.** RCP8.5 SSP5 high-end sea level rise, for four cases of adaptation. For region abbreviations, please see Supplementary Table 1.

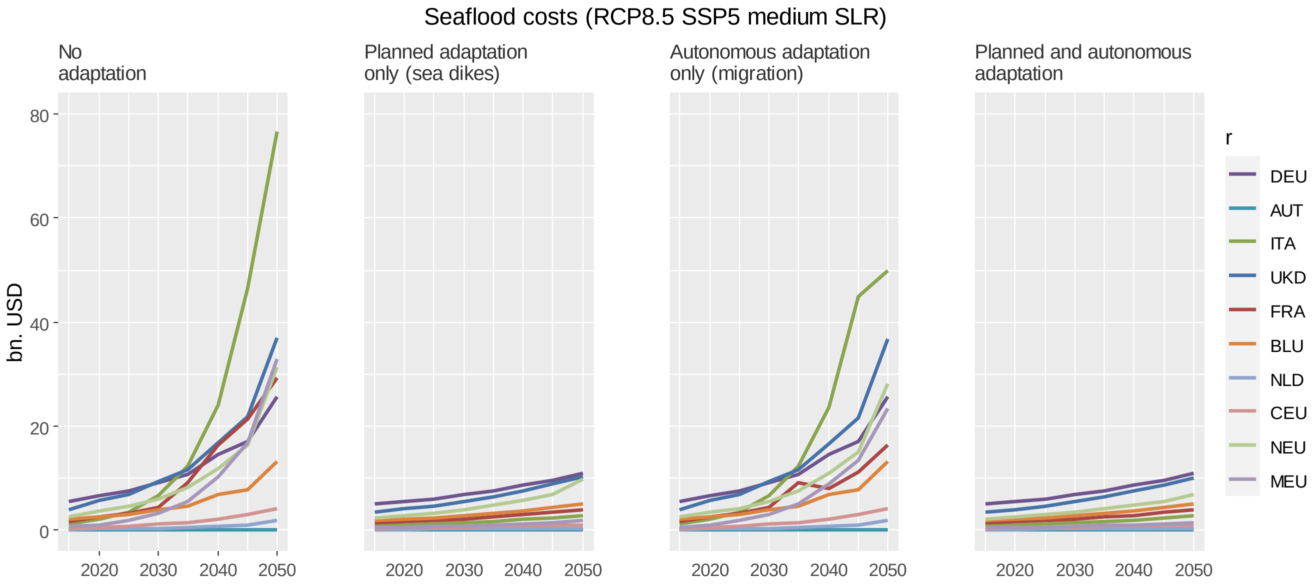

Supplementary Figure 17. **Direct sea flood costs for European regions.** RCP8.5-SSP5 medium sea level rise, for four cases of adaptation. For region abbreviations, please see Supplementary Table 1.

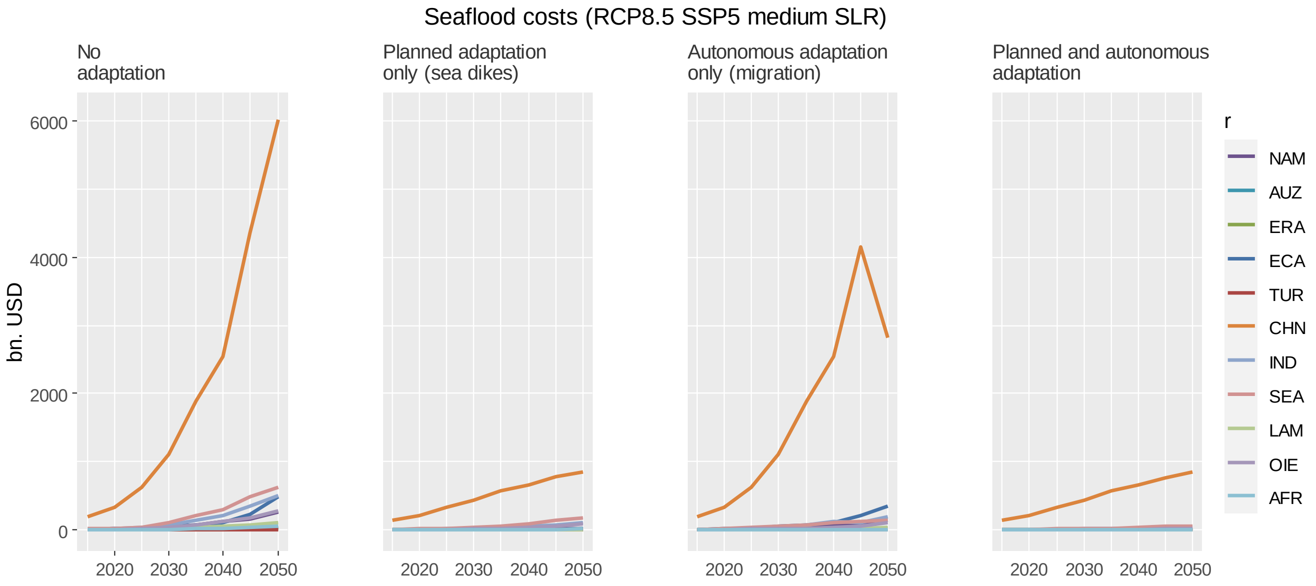

Supplementary Figure 18. **Direct sea flood costs for Rest of the World.** RCP8.5-SSP5 medium sea level rise, for four cases of adaptation. For region abbreviations, please see Supplementary Table 1.

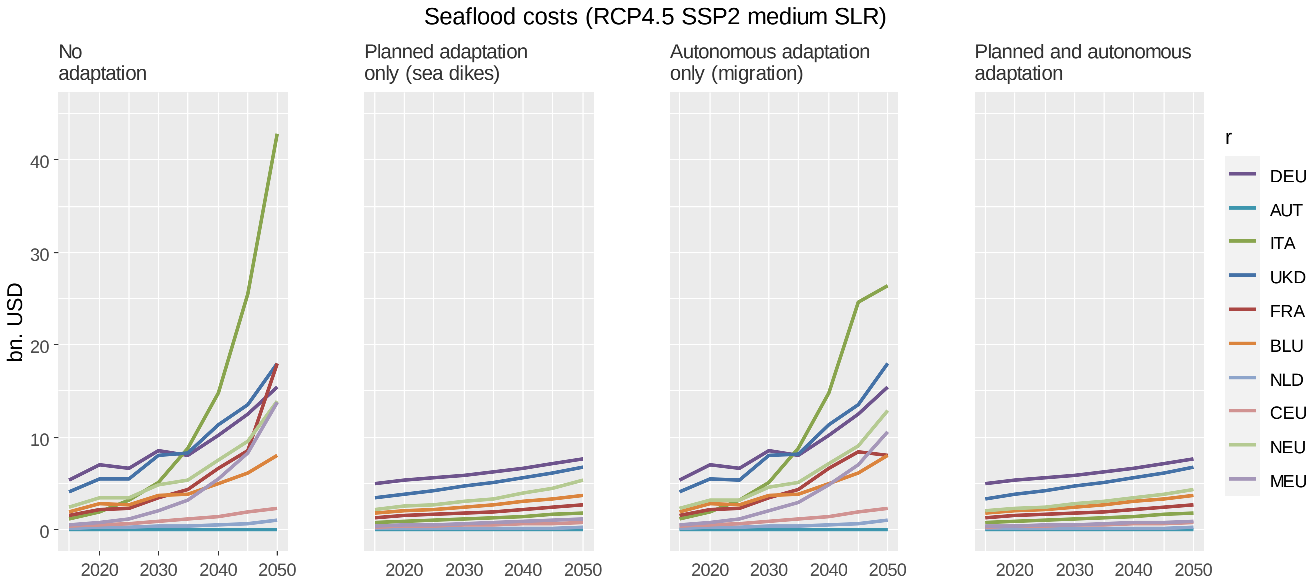

Supplementary Figure 19. **Direct sea flood costs for European regions.** RCP4.5-SSP2 medium sea level rise, for four cases of adaptation. For region abbreviations, please see Supplementary Table 1.

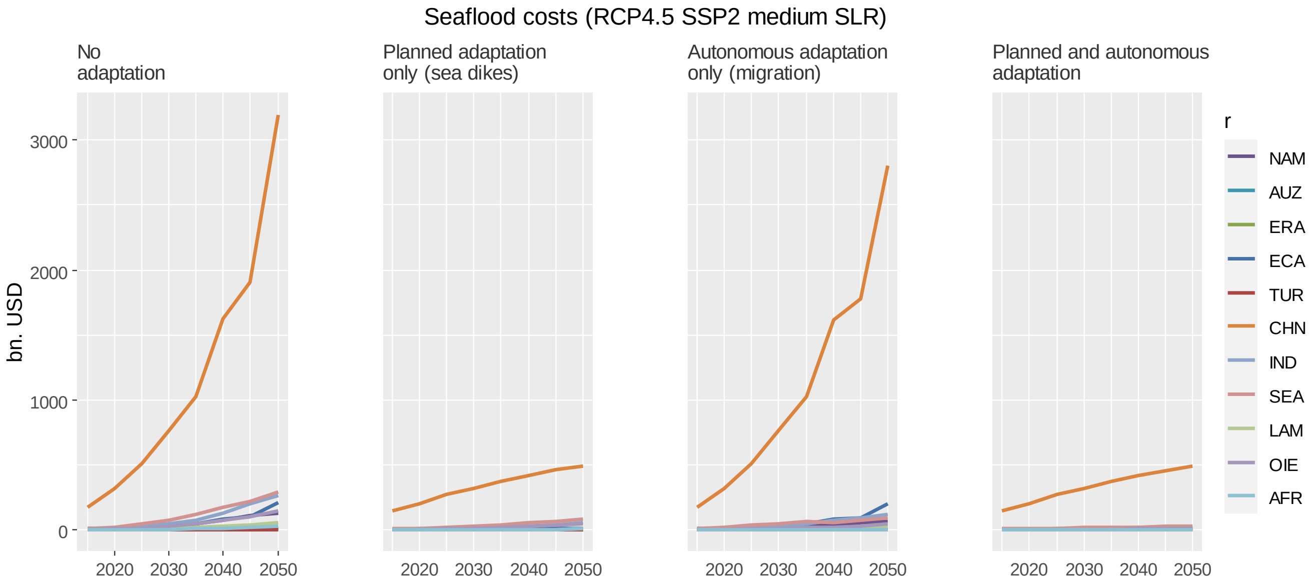

Supplementary Figure 20. **Direct sea flood costs for Rest of the World.** RCP4.5-SSP2 medium sea level rise, for four cases of adaptation. For region abbreviations, please see Supplementary Table 1.

Migration costs

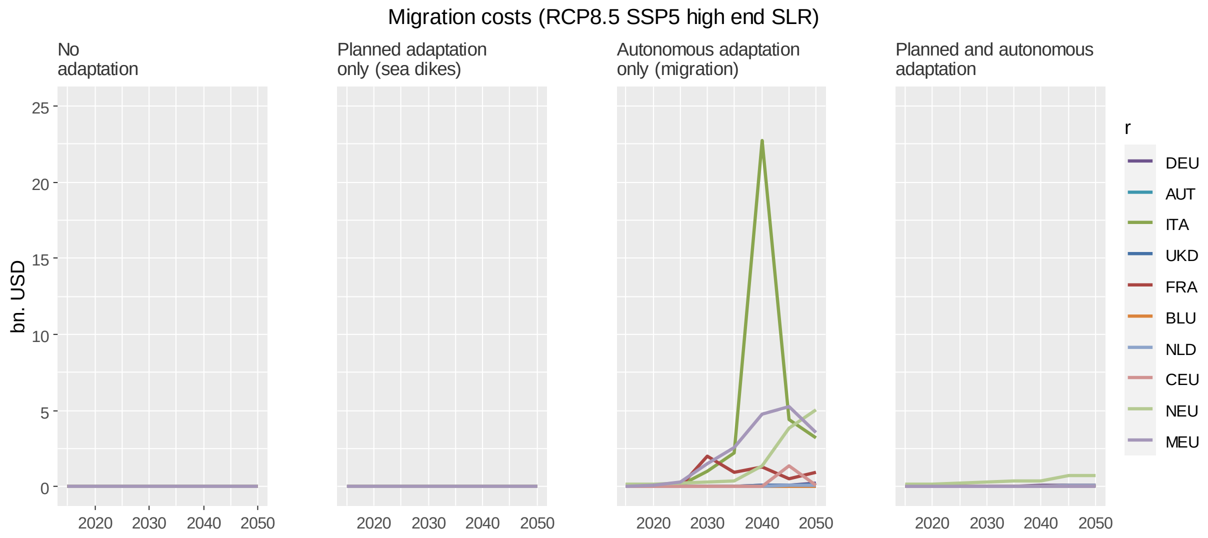

Supplementary Figure 21. **Direct migration costs for European regions.** RCP8.5 SSP5 high-end sea level rise, for four cases of adaptation. For region abbreviations, please see Supplementary Table 1.

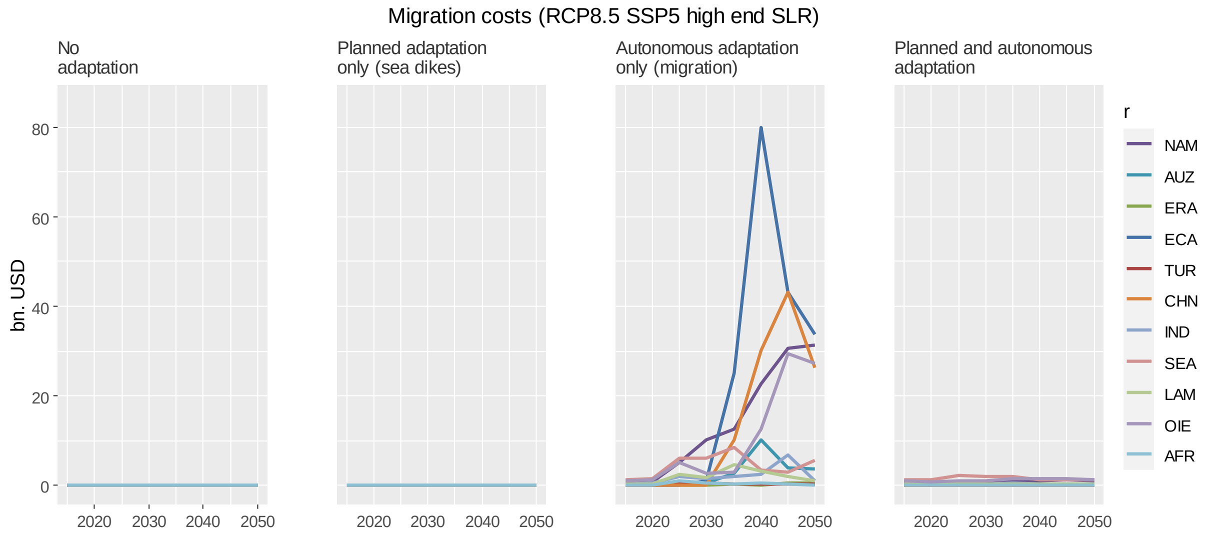

Supplementary Figure 22. **Direct migration costs for Rest of the World.** RCP8.5 SSP5 high-end sea level rise, for four cases of adaptation. For region abbreviations, please see Supplementary Table 1.

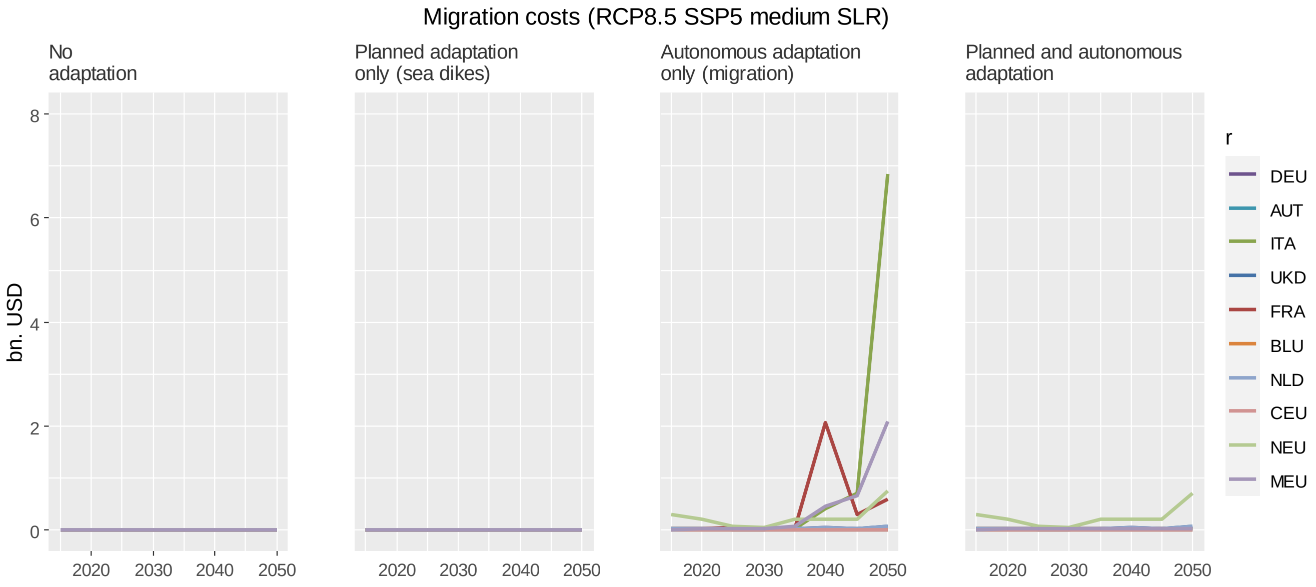

Supplementary Figure 23. **Direct migration costs for European regions.** RCP8.5 SSP5 medium sea level rise, for four cases of adaptation. For region abbreviations, please see Supplementary Table 1.

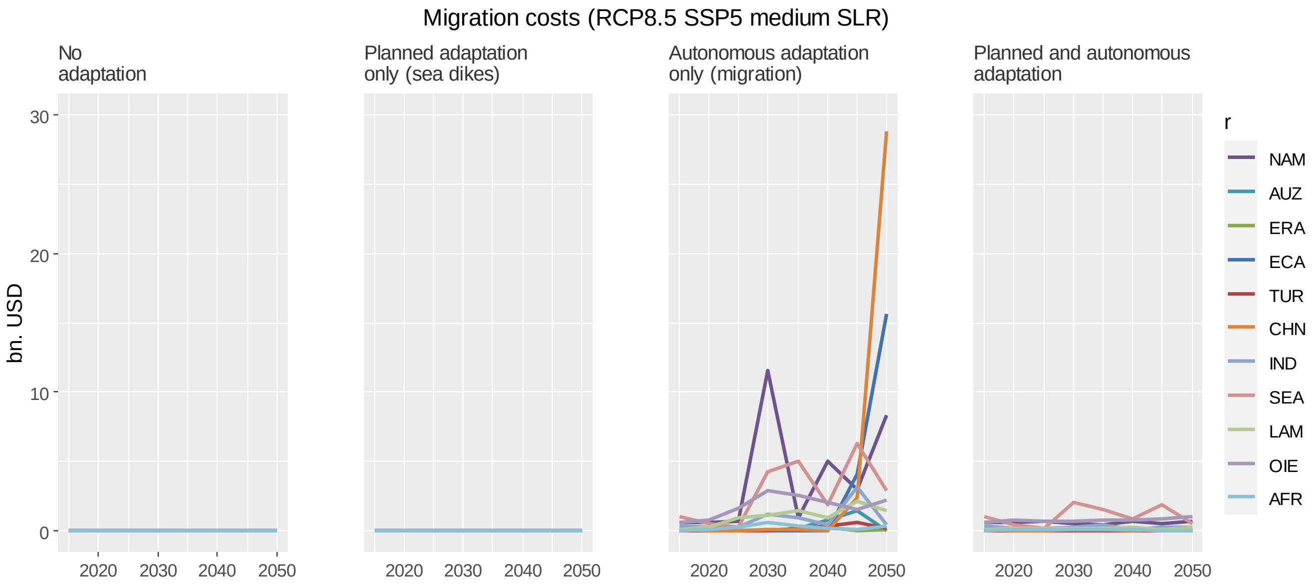

Supplementary Figure 24. **Direct migration costs for Rest of the World.** RCP8.5 SSP5 medium sea level rise, for four cases of adaptation. For region abbreviations, please see Supplementary Table 1.

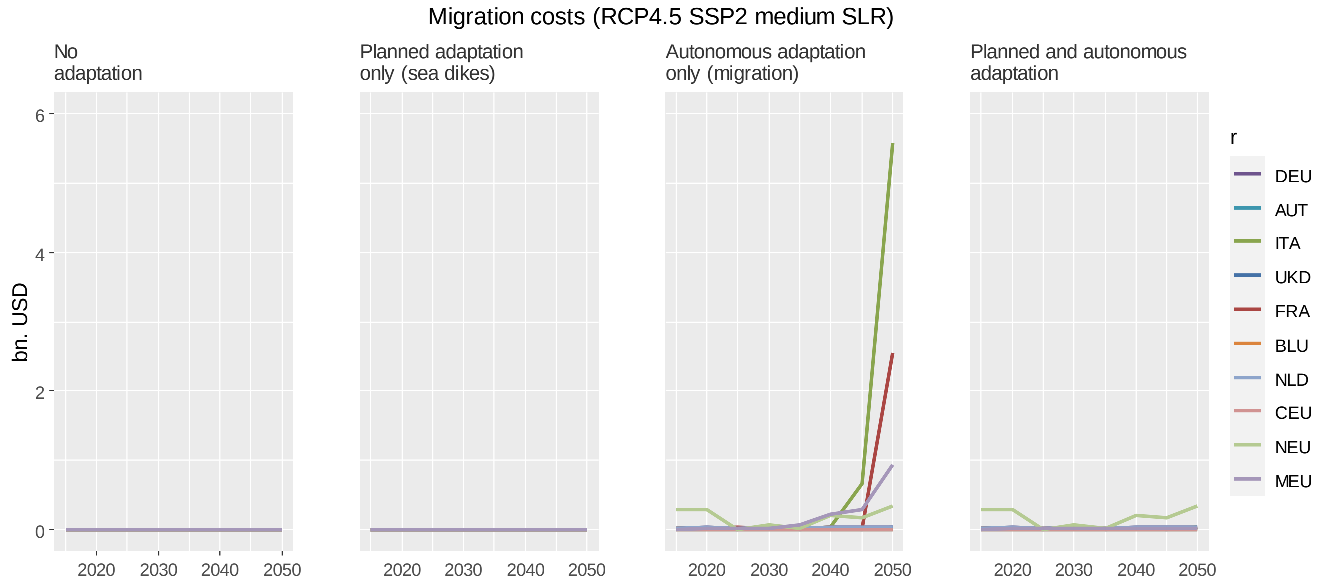

Supplementary Figure 25. **Direct migration costs for European regions.** RCP4.5 SSP2 medium sea level rise, for four cases of adaptation. For region abbreviations, please see Supplementary Table 1.

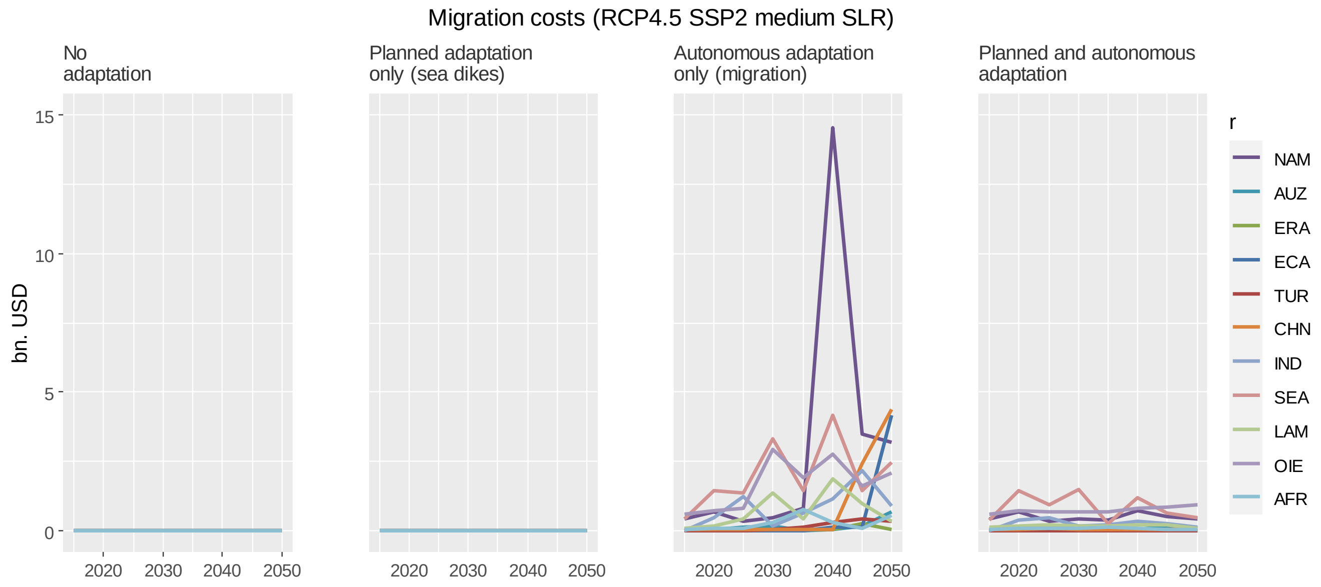

Supplementary Figure 26. **Direct migration costs for Rest of the World.** RCP4.5 SSP2 medium sea level rise, for four cases of adaptation. For region abbreviations, please see Supplementary Table 1.

Further results

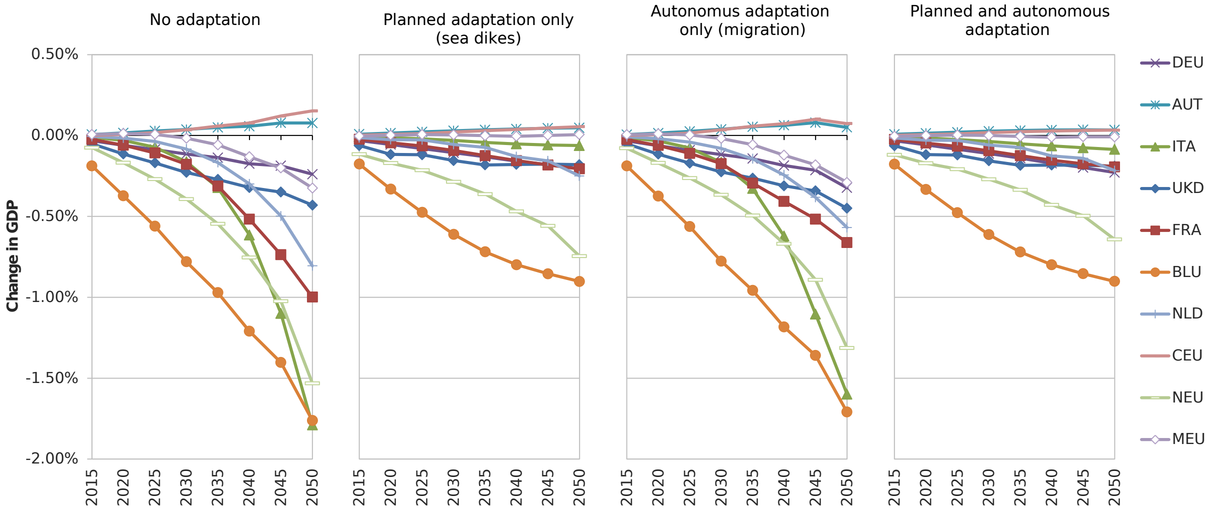

Supplementary Figure 27. **Change in GDP for European regions.** RCP8.5-SSP5 medium sea level rise, relative to Baseline scenario, for four cases of adaptation. For region abbreviations, please see Supplementary Table 1.

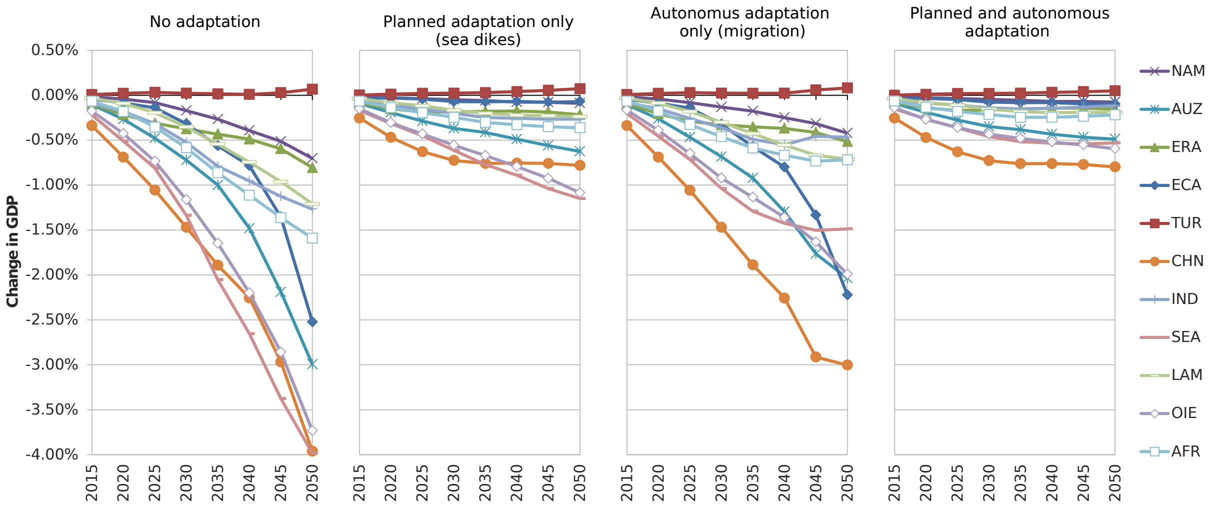

Supplementary Figure 28. **Change in GDP for ROW regions.** RCP8.5-SSP5 medium sea level rise, relative to Baseline scenario, for four cases of adaptation. For region abbreviations, please see Supplementary Table 1.

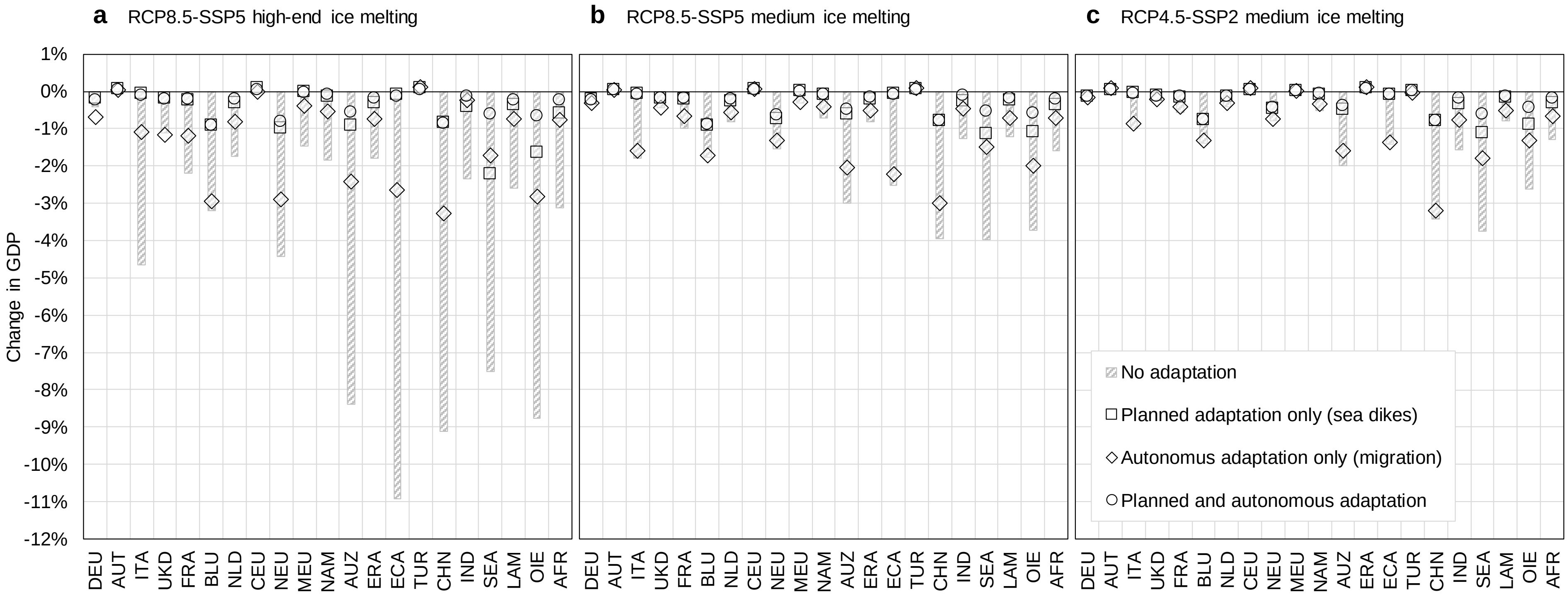

Supplementary Figure 29. **Change in GDP for four cases of adaptation relative to baseline scenario. a** RCP8.5-SSP5 high-end sea level rise. **b** RCP8.5-SSP5 medium sea level rise. **c** RCP4.5-SSP2 medium sea level rise. For region abbreviations, please see Supplementary Table 1.

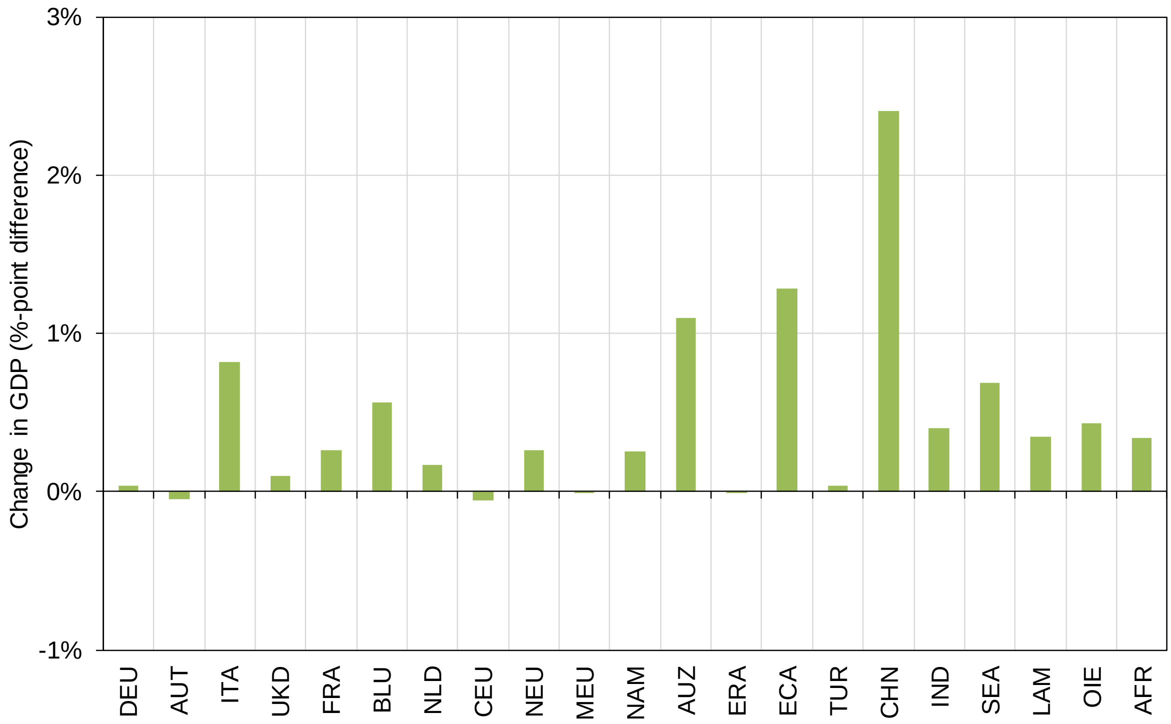

Supplementary Figure 30. **Macroeconomic effectiveness.** Comparison between planned adaptation (sea dikes) and autonomous adaptation (migration) under RCP4.5-SSP2 in 2050 under medium sea level rise. Effectiveness is measured as %-point difference of GDP loss between planned adaptation (sea dikes) and autonomous adaptation (migration). Positive bars indicate that GDP losses are less severe with planned adaptation only and vice versa.

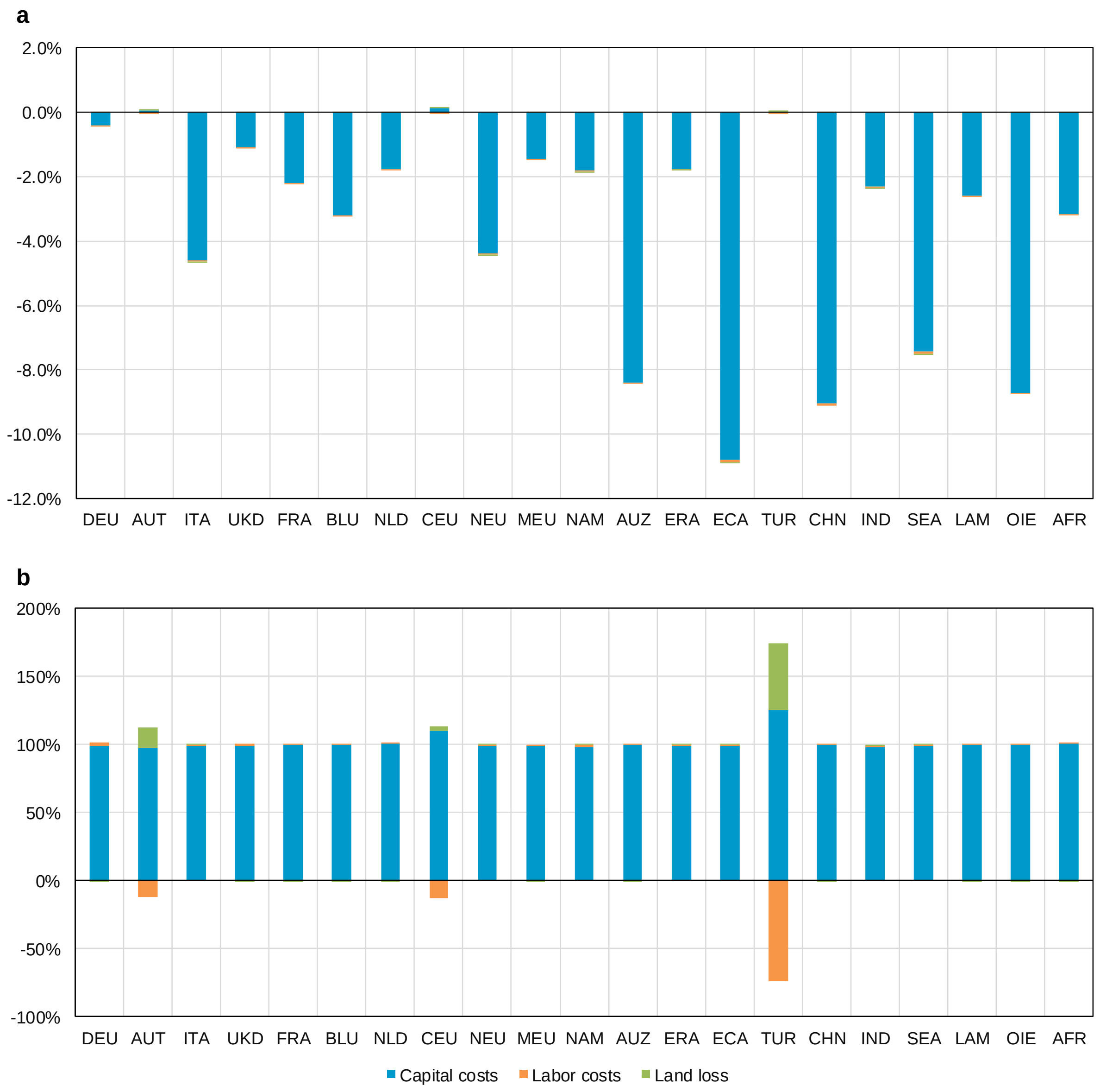

Supplementary Figure 31. **Sensitivity analysis on the contribution of different cost channels.** RCP8.5 SSP5 high-end sea level rise with no adaptation. Positive values mean that the respective impact channel in isolation contributes positively to GDP loss, negative values indicate that the impact channel would lead to GDP gains. For all regions, the sum of all impact channels' contributions is 100%. **a** decomposition of impact channels for relative GDP loss by region. **b** relative contribution of impact channels to GDP loss. Region abbreviations: DEU: Germany; AUT: Austria; ITA: Italy; UKD: UK; FRA: France; BLU: Belgium and Luxemburg; NLD: Netherlands; CEU: Central EU 27 + Switzerland; NEU: Northern EU 27+ Liechtenstein, Norway and Iceland; MEU: Mediterranean and South-eastern EU 27; NAM: North America; AUZ: Australia and New Zealand; ERA: Eurasian countries; ECA: Emerging economies- Asia; TUR: Turkey; CHN: China; IND: India; SEA: South-East Asia; LAM: Latin America (w/o Venezuela); OIE: Oil exporting countries (OPEC: Middle East and Africa + Venezuela); AFR: Africa

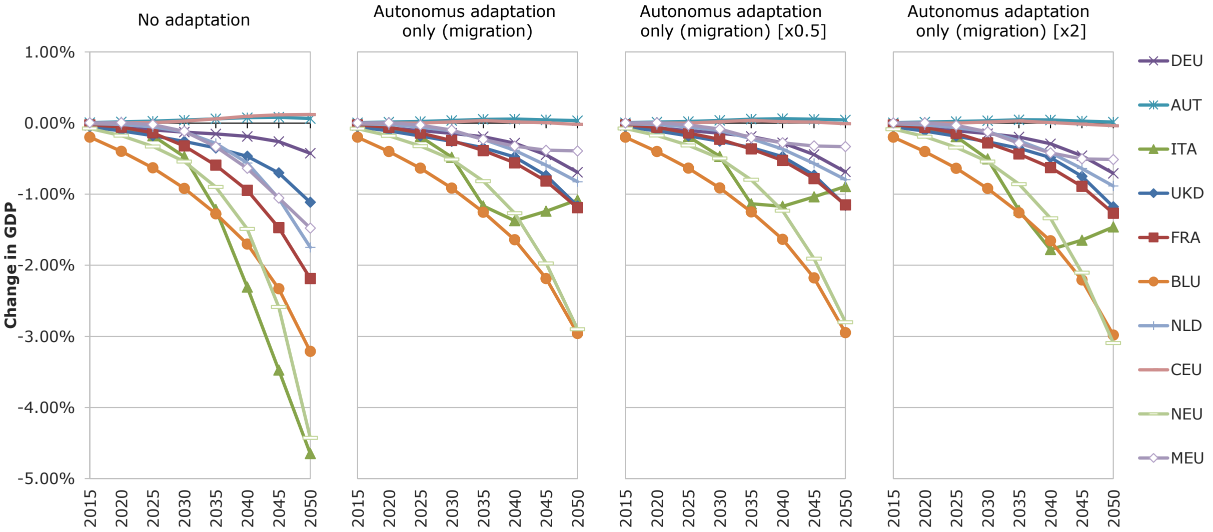

Supplementary Figure 32. **GDP effects for European regions with variation of migration costs.** Results under RCP8.5-SSP5 high-end sea-level rise, relative to baseline scenario, for the cases of no-adaptation, autonomous-adaptation-only, as well as autonomous-adaptation-only with lower (halved, x0.5) and higher (doubled, x2) migration costs per migrant. For region abbreviations, please see Supplementary Table 1.

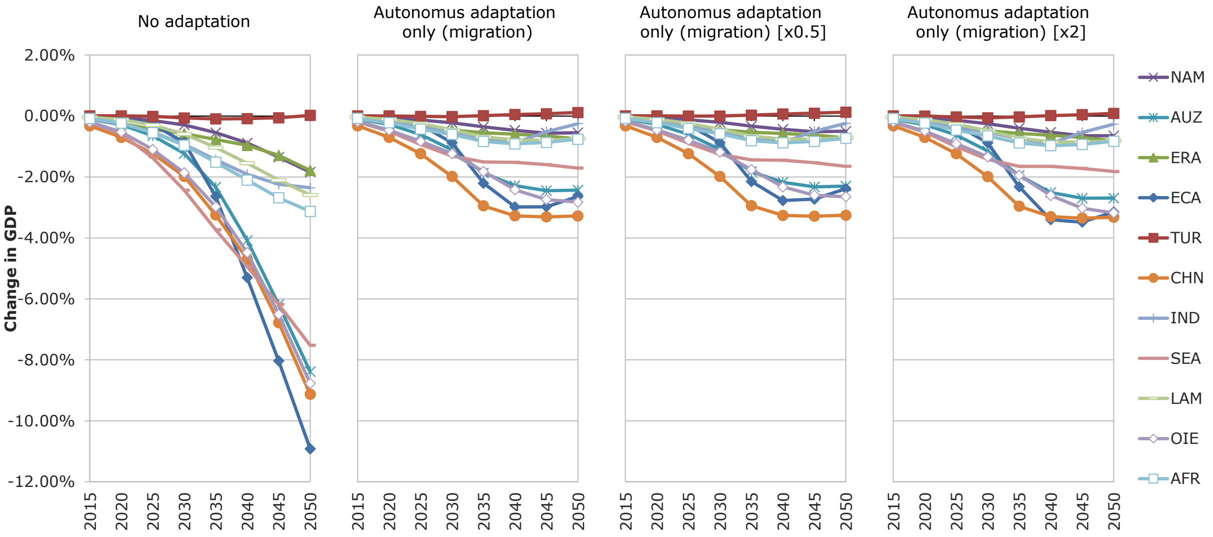

Supplementary Figure 33. **GDP effects for ROW regions with variation of migration costs.** Results under RCP8.5-SSP5 high-end sea-level rise, relative to baseline scenario, for the cases of no-adaptation, autonomous-adaptation-only, as well as autonomous-adaptation-only with lower (halved, x0.5) and higher (doubled, x2) migration costs per migrant. For region abbreviations, please see Supplementary Table 1.

	2050			2100		
	RCP8.5-SSP5 (high end)	RCP8.5-SSP5 (med)	RCP4.5-SSP2	RCP8.5-SSP5 (high end)	RCP8.5-SSP5 (med)	RCP4.5-SSP2
**2015**	0.39	0.19	0.16	1.62	0.63	0.45
**1985-2005**	0.46	0.24	0.21	1.7	0.7	0.5

Supplementary Table 2. **Sea level rise in m across scenarios and different reference periods.**

References

1. Schinko, T., Bednar-Friedl, B., Steininger, K. W. & Grossmann, W. D. Switching to carbon-free production processes: Implications for carbon leakage and border carbon adjustment. *Energy Policy* **67**, 818–831 (2014).
2. Peters, J. C. The GTAP-Power Data Base: Disaggregating the Electricity Sector in the GTAP Data Base. *J. Glob. Econ. Anal.* **1**, 209–250 (2016).
3. Armington, P. S. A Theory of Demand for Products Distinguished by Place of Production (Une theorie de la demande de produits differencies d'apres leur origine) (Una teoria de la demanda de productos distinguiendolos segun el lugar de produccion). *Staff Pap. - Int. Monet. Fund* **16**, 159 (1969).
4. O'Neill, B. C. *et al.* A new scenario framework for climate change research: the concept of shared socioeconomic pathways. *Clim. Change* **122**, 387–400 (2014).
5. World Bank. Gross savings (% of GDP) | Data. https://data.worldbank.org/indicator/NY.GNS.ICTR.ZS (2019).
6. Dai, H.-C., Zhang, H.-B. & Wang, W.-T. The impacts of U.S. withdrawal from the Paris Agreement on the carbon emission space and mitigation cost of China, EU, and Japan under the constraints of the global carbon emission space. *Adv. Clim. Change Res.* **8**, 226–234 (2017).
7. IEA. *World Energy Outlook 2018*. (Inernational Energy Agency, 2018).
8. Fricko, O. *et al.* The marker quantification of the Shared Socioeconomic Pathway 2: A middle-of-the-road scenario for the 21st century. *Glob. Environ. Change* **42**, 251–267 (2017).
9. Mayer, J., Bachner, G. & Steininger, K. W. Macroeconomic implications of switching to process-emission-free iron and steel production in Europe. *J. Clean. Prod.* **210**, 1517–1533 (2019).
10. Koesler, S. & Schymura, M. Substitution elasticities in a Constant Elasticity of Substitution framework - empirical estimates using nonlinear least squares. *Econ. Syst. Res.* **27**, 101–121 (2015).
